# Acinic Cell Carcinoma of the Nasal Lateral Wall

**DOI:** 10.7759/cureus.18748

**Published:** 2021-10-13

**Authors:** Sílvia Dionísio, Eduardo Ventura, Joana Gonçalves, Rafael Nobre, Hugo Marques

**Affiliations:** 1 Maxillofacial Surgery Department, Centro Hospitalar e Universitário do Porto, Porto, PRT

**Keywords:** epistaxis, nasal cavity, nasal tumor, nasal mass, acinic cell carcinoma

## Abstract

Primary acinic cell carcinoma arising in the nose is exceptionally rare. In this report, we present a unique case of an acinic cell carcinoma of the nasal lateral wall, and it is only the second such case to be reported. We also engage in a systematic review of all 18 cases of acinic cell carcinoma of the nose reported in the literature in English so far.

## Introduction

Acinic cell carcinoma is an uncommon salivary tumor, which arises most frequently in the parotid gland and rarely in the minor salivary glands [1]. Most of the acinic cell carcinomas originating in minor salivary glands have been found in the oral cavity [1]. Primary acinic cell carcinoma arising in the nose is exceptionally rare, and it is thought to account for only 1-4% of all malignant nasal neoplasms [2]. Only 18 cases of acinic cell carcinoma arising in the nasal cavity have been previously reported in the literature in English [3].

## Case presentation

A 62-year-old male patient presented to our emergency room with a history of right nasal mass for 10 years. The patient reported occasionally experiencing epistaxis and in the last six months, there had been a slow, gradual growth. However, it had not been noticed during that period.

**Figure 1:**
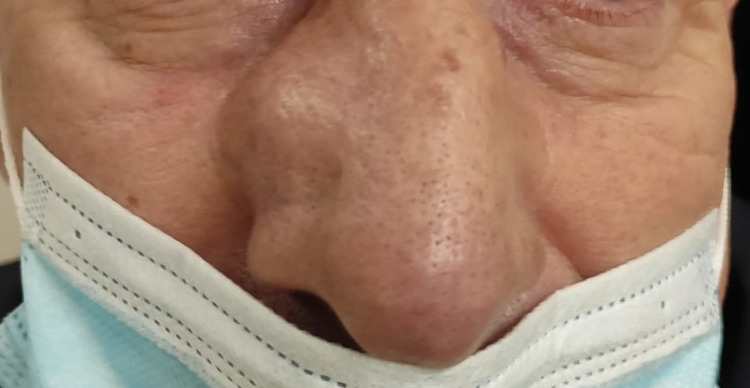
Image showing a round mass on the right-side wall of the nose

**Figure 2:**
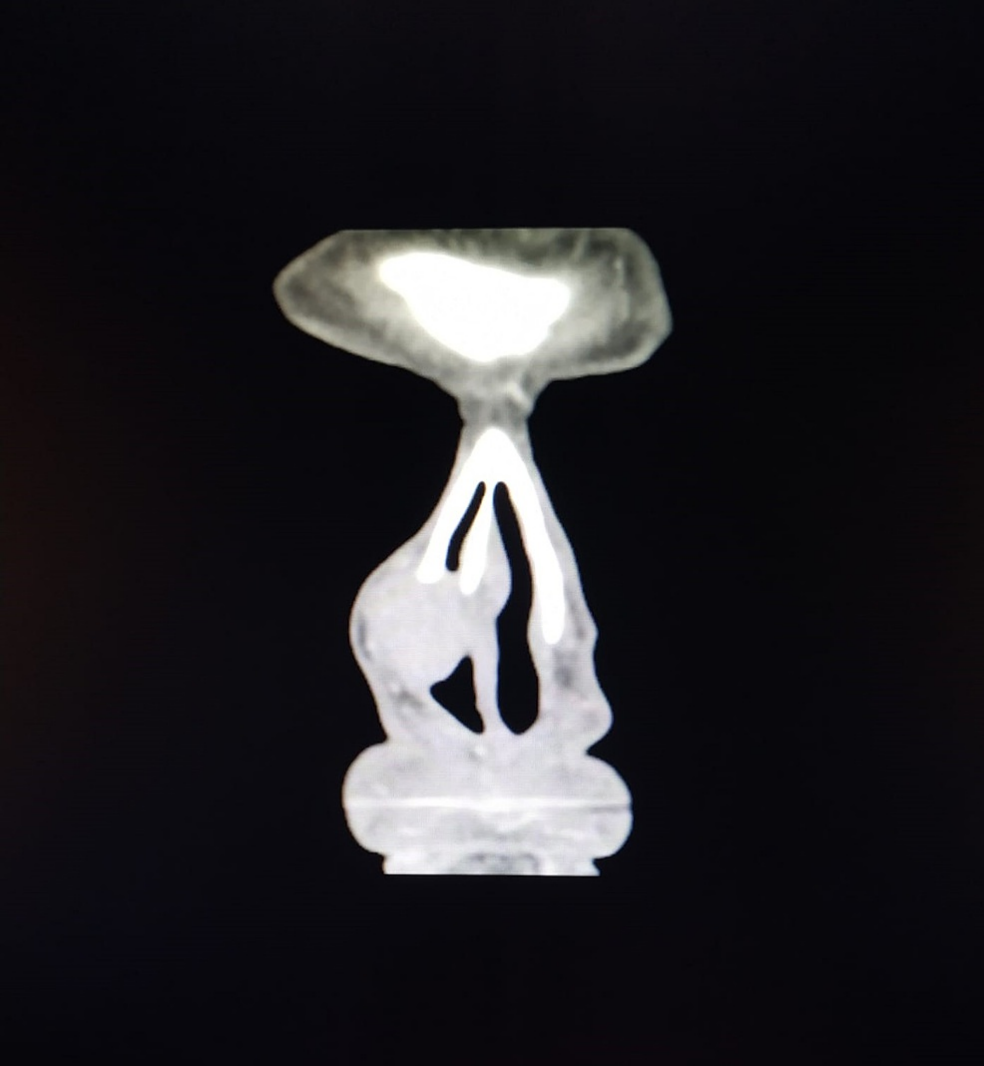
CT scan - coronal view CT scan showing a homogeneous nodular mass of soft tissue with the erosion of the frontal maxillary apophysis extending into the nasal vestibule CT: computed tomography

**Figure 3:**
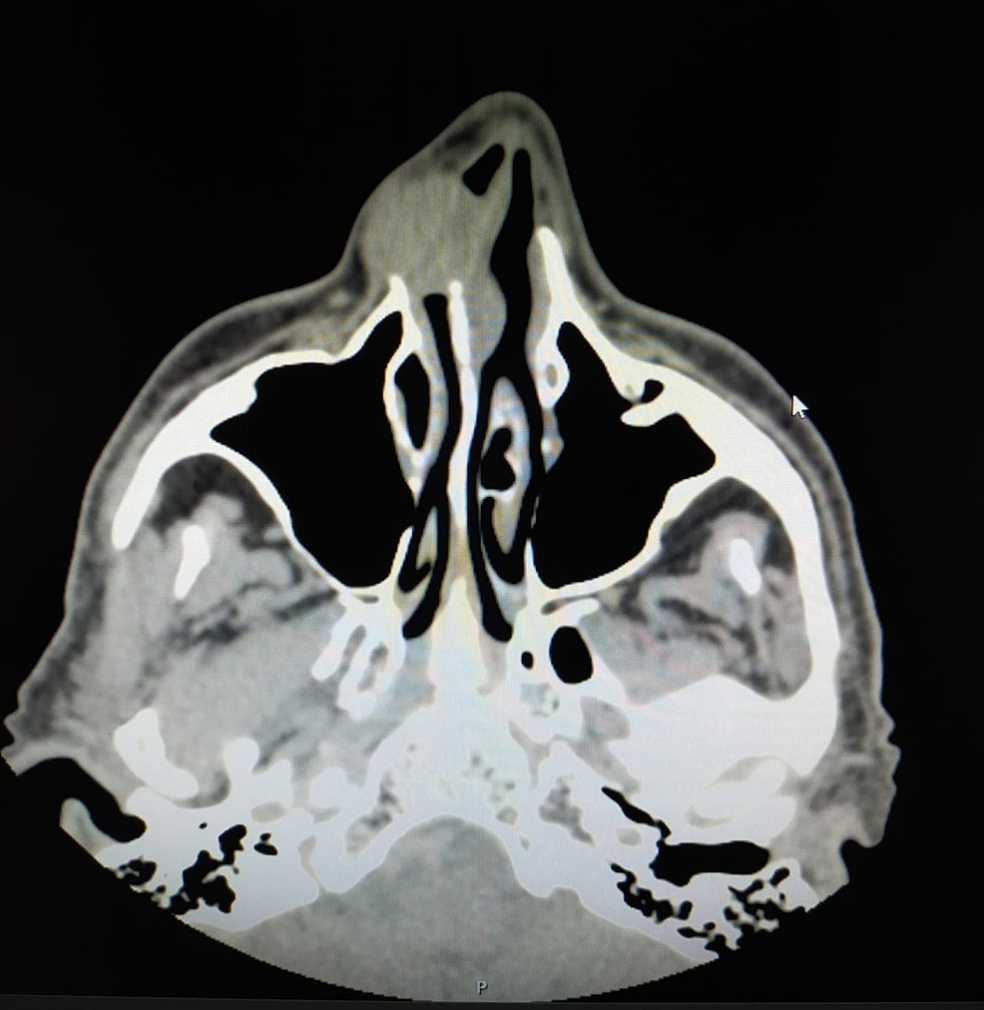
CT scan - axial view CT scan showing a homogeneous nodular mass of soft tissue with the erosion of the frontal maxillary apophysis extending into the nasal vestibule CT: computed tomography

He had a round, soft, well-defined mass on the lateral nasal wall (Figure 1). An ultrasound revealed a solid oval lesion with some internal vascularization in the right wing of the nose, measuring 15 x 11 x 19 mm, which apparently caused an interruption of the cortical bone. For additional characterization of the lesion, a CT scan was performed. The CT scan showed a homogeneous nodular structure of soft tissue, centered on the region of the frontal maxillary apophysis on the right, with its erosion, and extending into the nasal vestibule. The structure measured approximately 17-18 mm in diameter; it had smooth, regular contours, and the adjacent bone limit was molded, constituting characteristics suggestive of benignity (Figures 2, 3).

The surgical removal of the mass was performed. The macroscopic findings during the procedure suggested that the primary site of the mass was the right lateral wall, with expansion to the vestibule and without invasion of the nasal septum. The mass was totally excised along with the area of the surrounding skin. The postoperative period was uneventful and the patient was discharged on the same day.

**Figure 4:**
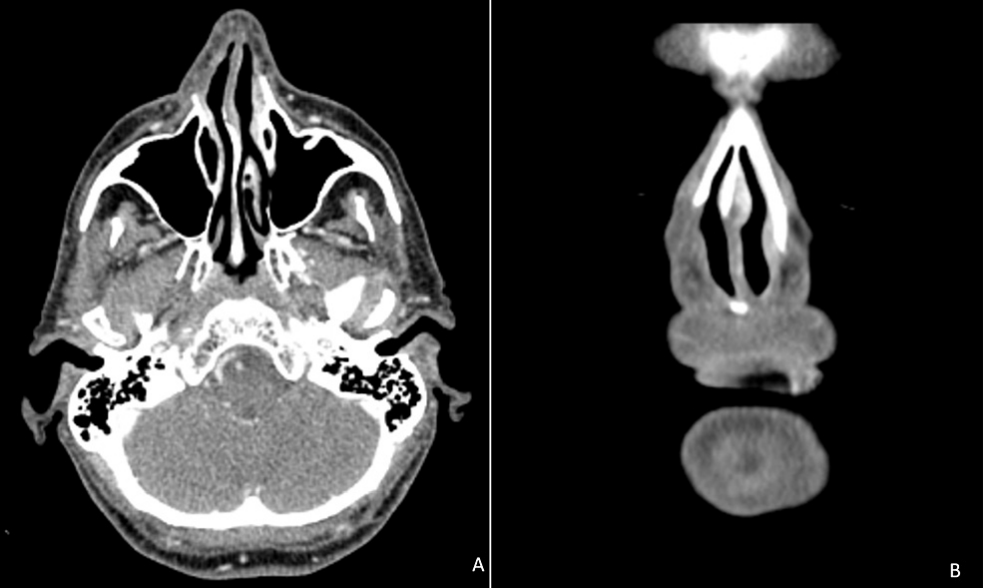
CT scans showing no signs of local recurrence A: axial view; B: coronal view CT: computed tomography

Microscopically, the tumor had the appearance of classic acinic cell carcinoma. Necrosis and mitosis were absent. The neoplasm was intersected by the margins (fulguration). Given the unexpected histological diagnosis, a staging CT was performed, which showed a total excision of the lesion and no signs of local recurrence or metastasis. It was decided that the patient should undergo radiotherapy by the oncology department. At the follow-up six months later, the patient had no signs of recurrence (Figure 4).

## Discussion

Acinic cell carcinoma is a rare, slow-growing, low-grade malignancy tumor of the salivary gland [4]. Acinic cell carcinoma comprises only 0.3-0.5% of all minor salivary neoplasms and usually occurs in the oral cavity, and lesions arising in the nasal cavity are extremely rare [1]. There are a few reported cases in the literature in English; the previously reported cases are summarized in Table 1 [3-17].

**Table 1:** Previously reported cases of acinic cell carcinoma of the nose M: male; F: female; S: surgery; RT: radiotherapy; N/S: not specified

Author/references	Age in years/gender	Location	Symptoms	Treatment	Follow-up
Current case	62/M	Nasal lateral wall	Epistaxis	S + RT	6 months
Hou et al., 2017 [3]	63/F	Nasal septum	Obstruction, hyposmia, and epistaxis	S	2 years
Gangadhara Somayaji et al., 2014 [5]	65/F	Nasal lateral wall	Obstruction, epistaxis	S	1 year
Hammami et al., 2010 [4]	47/F	Nasal septum	Obstruction, hyposmia	S + RT	4 years
Manganaris et al., 2010 [6]	51/F	Vestibule	Pain	S	3 years
Neto et al., 2005 [7]	60/F	Superior meatus	Obstruction	S	17 years
Neto et al., 2005 [7]	42/F	Inferior turbinate	Obstruction	S	7 years
Neto et al., 2005 [7]	65/M	Inferior turbinate	Obstruction	S	4 years
Neto et al., 2005 [7]	50/M	Nasal cavity	Obstruction	S + RT	12 years and recurrence
Sapçi et al., 2000 [8]	47/M	Nasal septum	Obstruction, epistaxis	S	1.5 years
Jasin et al., 1999 [9]	44/F	Nasal septum	N/S	S	2.5 years
Von Biberstein et al., 1999 [10]	76/F	Middle turbinate	Nasal polyp	S	3 years
Schmitt et al., 1994 [11]	60/M	Inferior turbinate	Obstruction	N/S	N/S
Valerdiz-Casasola et al., 1993 [12]	47/M	Nasal cavity	Obstruction, epistaxis	S + RT	10 months
Takimoto et al., 1989 [13]	60/F	Middle and inferior turb	Nasal polyp, epistaxis	S	2 years
Hanada et al., 1988 [14]	68/M	Inferior turbinate	Obstruction	S + RT	3 years
Finkelhor et al., 1987 [15]	45/F	Nasal septum	Obstruction	S	N/S
Ordonez et al., 1986 [16]	60/F	Superior meatus	Nasal polyp, epistaxis	S	7 years
Perzin et al., 1981 [17]	75/F	Inferior turbinate	Obstruction, epistaxis	S	N/S

The mean age of the patients was 57.2 years (range: 42-76 years). Women constituted the majority of the patients (63%). Nine cases developed on turbinate, five were located on the nasal septum, two on the nasal cavity and nasal lateral wall, and only one case was reported on the vestibule. The most common presenting complaint was nasal obstruction (84%), followed by epistaxis (42%) and hyposmia (10.5%). There were no tumor markers or imaging characteristics that allowed for a preoperative diagnosis, and all cases were diagnosed after surgical excision. The diagnoses were based entirely on histopathological evaluations [3].

Acinic cell carcinomas should be treated by complete surgical excision and the prognosis is related to tumor extension and the quality of resection [3,5,17]. Postoperative radiation is not routinely advocated for these low-grade salivary malignancies but may be used for tumors with positive surgical excision margins, tumors with extensive perineural and/or lymphovascular invasion, advanced or high-grade tumors, or recurrent tumors [18]. Chemotherapy for acinic cell carcinoma is considered ineffective [3]. Elective neck dissection is not warranted, because the incidence of cervical nodal metastasis is relatively low, with the reported nodal metastasis rates for parotid acinic cell carcinomas being 5-10% [2,17,18].

The treatment in all cases was surgery, and in five cases, patients underwent radiotherapy as well. To date, recurrence has been reported in only one case.

## Conclusions

Acinic carcinoma is rarely located in the nasal cavity. Its nasal lateral wall origin is highly unusual. Curative treatment is surgery alone or surgery associated with radiotherapy. Patients with acinic cell carcinoma should be followed up for long periods, because recurrence may occur many years after the treatment. However, further studies with longer follow-up periods are needed to gain deeper insight into the clinical behavior of nasal acinic cell carcinoma.
